# Supplementary material for: Lantox—The Chinese Botulinum Toxin Drug—Complete English Bibliography and Comprehensive Formalised Literature Review
Source: Toxins (Basel). 2021 May 22;13(6):370. doi: 10.3390/toxins13060370 (PMC8224638; doi:10.3390/toxins13060370)
Supplement: Supplementary file 1 [file toxins-13-00370-s001.zip › toxins-1172173 supplementary for final.pdf]

Supplementary Materials: Lantox—The Chinese Botulinum Toxin Drug—Complete English Bibliography and Comprehensive Formalised Literature Review

Dirk Dressler, Lizhen Pan, Junhui Su, Fei Teng and Lingjing Jin

Blepharospasm

Chen GZ, Gao C (2001) (OS) Treatment of blepharospasm and hemifacial spasm with botulinum toxin A [in Chinese]. Chin J Optom Ophthalmol 3:182-183.
Chen J, Lu YQ, Kang ML (2002) (OS) Blepharospasm treated with botulinum toxin A [in Chinese]. Chin Rem Clin 2:145-147.
Chen XH, Zhuang ZH (2006) (OS) Effects of botulinum toxin A on idiopathic blepharospasm and facial spasm [in Chinese]. Chin J Pract Nerv Dis 9:15-16.
Fan YH (2010) (OS) Clinical observations on botulinum toxin A therapy for blepharospam and hemifacial spasm [in Chinese]. Chin J Aesthetic Med 19:759.
Hu XY, Cai HY, Shao YQ (2004) (OS) Effects and dosages of botulinum toxin A for long-term treatment of idiopathic blepharospasm [in Chinese]. Chin J New Drugs Clin Rem 23:40-42.
Jiao YH, Wang YD, Pan Z (2014) (OS) Clinical study on botulinum toxin A for blepharospasm [in Chinese]. Int Eye Sci 14:1350-1351.
Kang XL, Gan CZ (2008) (OS) Clinical study of botulinum toxin A treatment of blepharospasm and hemifacial spasm [in Chinese]. Chin J Pract Nerv Dis 11:15-16.
Lan ZH, Zhang LZ (2001) (OS) Clinical observations on botulinum toxin A therapy of 364 cases of blepharospasm and hemifacial spasm [in Chinese]. Chin J Strabismus Pediatr Ophthalmol 9:158-159.
Li HM (2001) (OS) Treatment of blepharospasm and hemifacial spasm with botulinum toxin A [in Chinese]. J Pract Aesth Plasti Surg 12:194.
Li W (1998) (OS) Botulinum toxin A for the treatment of blepharospasm [in Chinese]. Chin J Pract Ophthalmol 16:189-190.
Liao Q, Li C, Liu X (2011) (OS) Clinical observation on botulinum toxin A on patients with blepharospasm [in Chinese]. J Clin Ophthalmol 19:80-81.
Liu MJ (2008) (OS) Clinical analysis of botulinum toxin A in the treatment of blepharospasm [in Chinese]. Chin J Clin Pharmacol 24:103,146.
Lou XB, Dai S, Li YZ, Liu XY (2005) (OS) Clinical use of botulinum toxin A in blepharospasm and strabismus [in Chinese]. Chin J Strabismus Pediatr Ophthalmol 13:173-174.
Lu XL, Cai CY, Tu HF (2016) (RCT) Effects of different injection sites of botulinum toxin A in the treatment of blepharospasm [in Chinese]. Int Eye Sci 16:2351-2353.
Luo ZR, Kang QY (2004) (OS) Causes and management of complications of botulinum toxin A for essentiaI blepharospasm and Meige syndrome [in Chinese]. Int J Ophthalmol 4:478-480.
Quagliato EM, Carelli EF, Viana MA (2010) (RCT) Prospective, randomised, double-blind study comparing botulinum toxins type a Botox and Prosigne for blepharospasm and hemifacial spasm treatment. Clin Neuropharmacol 33:27-31.
Rieder CR, Schestatsky P, Socal MP, Monte TL, Fricke D, Costa J, Picon PD (2007) (IS) A double-blind, randomised, crossover study of prosigne versus botox in patients with blepharospasm and hemifacial spasm. Clin Neuropharmacol 30:39-42.
Tong Y, Xu TH, Fan YX (1999) (OS) Botulinum toxin A for blepharospasm and hemifacial spasm [in

Chinese]. J Clin Ophthalmol 7:330-331.
Wang J, Fei XQ, Zhao LL, Lu W (2004) (OS) Treatment of blepharospasm and hemifacial spasm with botulinum toxin A: clinical analysis of 657 cases [in Chinese]. Chin J Strabismus Pediatr Ophthalmol 12:37-38.
Wang JL, Zhang X (2006) (OS) Efficacy of repeated botulinum toxin A injections on blepharospasm and hemifacial spasm [in Chinese]. J Clin Otorhinolaryngol China 20:292-294.
Wu CJ, Shen JH, Chen Y, Lian YJ (2011) (IS) Comparison of two different formulations of botulinum toxin A for the treatment of blepharospasm and hemifacial spasm. Turk Neurosurg 21:625-629.
Wu J (2002) (OS) Treatment of idiopathic blepharospasm with botulinum toxin A [in Chinese]. Rec Adv Ophthalmol 22:127-128.
Xian WG (2003) (OS) Treatment of idiopathic blepharospasm and hemifacial spasm with botulinum toxin A [in Chinese]. China Trop Med 3:47-48.
Xiang YH, Fu Y (2008) (OS) The effect of botulinum toxin A in treatment of blepharospasm and hemifacial spasm [in Chinese]. J Clin Clin Exp Med 7:98-99.
Xu TH, Tong Y, Fan YX (2001) (OS) Clinical study on botulinum toxin A therapy of blepharospasm and Meige syndrome [in Chinese]. J Clin Ophthalmol 19:415-416.
Xu XH, Xu K, Peng Q, Xue YC, Pan XF (2016) (IS) Comparison of botulinum toxin A and complete resection of the periorbital muscle for idiopathic blepharospasm [in Chinese]. Int Eye Sci 16:1962-1964.
Yang H, Ding XH, Zhao XJ, Yin XF, Yi CX (2013) (IS) Comparison of different botulinum toxin A injection patterns in the treatment of blepharospasm [in Chinese]. Chin J Ophthalmol Otorhinolaryngol 13:236-238,242.
Yin XX, Yang XG, Zhu SL, Chu MF (2005) (OS) The clinical effects of botulinum toxin A therapy of blepharospasm and hemifacial spasm [in Chinese]. Chin J Pract Ophthalmol 23:1128-1159.
You SD, Liu FH (2011) (OS) Clinical observation of botulinum toxin A in 60 patients with blepharospasm [in Chinese]. Chin J Pract Nerv Dis 14:87-88.
Zeng L (2012) (OS) Efficacy of botuliunum toxin A in the treatment of blepharospasm and hemifacial spasm [in Chinese]. Chin J Strabismus Pediatr Ophthalmol 20:151-153,156.
Zhang X, Qu Y, Li JQ (2004) (OS) Clinical effects of botulinum toxin A on blepharospasm and Meige syndrome [in Chinese]. J Shandong Univ (Health Sci) 42:496.
Zhao J (2002) (OS) Treatment of blepharospasm and hemifacial spasm with botulinum toxin A [in Chinese]. Ophthalmol China 11:227-228.
Zhao SY (2009) (OS) Non-surgical treatment of 150 cases of blepharospasm and hemifacial spasm [in Chinese]. Chin J Optom Ophthalmol 11:232-233.
Zhao YJ, Yan JX, Li RC, Di BZ (1999) (IS) Botulinum toxin A for the treatment of blepharospasm in the elderly [in Chinese]. Chin J Gerontol 19:23-24.
Zhu DQ, Zhu T, Yu DC (2012) (IS) Botulinum toxin A combined with GABA drugs in blepharospasm and Meige syndrom [in Chinese]. J Apoplexy Nerv Dis 29:1036-1037.
Secondary Topic Publications
Qu F, Hu JH, Sun YJ, Shan LH, Yu P, Song Y (2003) (OS) Efficacy of botulinum toxin A in 156 patients with hemifacial spasm, blepharospasm and Meige syndrome [in Chinese]. J Apoplexy Nerv Dis 20:68-69.
Wang M, Yu C, Liu RY, Wang JF, Li DF (2002) (OS) Botulinum toxin A in 35 cases of hemifacial spasm, blepharospasm and Meige syndrome [in Chinese]. Clin Focus 17:518.
Xu J (2002) (OS) Clincial study on botulinum toxin A therapy of hemifacial spasm, blepharospasm and Meige syndrome [in Chinese]. J Guiyang Med Coll 27:40-41.
Meige Syndrome
Cheng DW, Wang F, Wang ZM (2016) (OS) Meige syndrome in 8 cases and review of literature [in

Chinese]. Chin J Aesth Plast Surg 27:362-364.
Cheng X, Yang JZ (2017) (OS) Botulinum toxin A treatment of Meige syndrome [in Chinese]. Chin J Integr Med Cardiocerebrovasc Dis 15:1540-1542.
Deng YF, Wang CL (2002) (OS) Botulinum toxin A in Meige syndrome and facial wrinkles [in Chinese]. Chin J Dermatol 35:393-395.
Deng YF, Zeng WS, Wang XH (2009) (OS) Efficacy of repeated botulinum toxin A treatment in Meige syndrome of the elderly [in Chinese]. Chin J Geriatr 28:584-586.
Fu YG (2011) (OS) Botulinum toxin A for treatment of Meige syndrome [in Chinese]. Clin Focus 16:400-401.
Hong W, Li CR, Yang XL, Ma MC, Ma HZ (2007) (OS) Botulinum toxin A in 21 patients with Meige syndrome [in Chinese]. Chin J Misdiagn 7:1832-1833.
Huang G, Tang XF (2002) (OS) Botulinum toxin A combined with Clonazepam and Benzhexol for the treatment of Meige syndrome [in Chinese]. J Clin Neurol 15:183-184.
Huang HQ, Chen KN, Chen L, Luo MK (2004) (OS) Efficacy of botulinum toxin A on Meige syndrome [in Chinese]. Chin J Clin Rehabil 8:7800
Huang HQ, Chen KN, Chen L, Luo MK (2005) (OS) Efficacy of botulinum toxin A in Meige syndrome. Chin J Clin Rehabil (Engl) 9:132-133.
Ou JB, Yao LQ, Ao LJ, Tang M (2014) (CS) Case report: botulinum toxin A in Meige syndrome [in Chinese]. Chin J Rehabil Med 29:871-872.
Song CL, Yao JH, Wang LF (2003) (OS) Botulinum toxin A in the treatment of Meige syndrome [in Chinese]. J Apoplexy Nerv Dis 20:259-260.
Wang J, Gao PF, Zhou FM, Xi GM (2011) (OS) Botulinum toxin A treatment of Meige syndrome: methods and results [in Chinese]. Chin J Aesthetic Med 20:191-193.
Wang SP, Wang CX (2003) (OS) Botulinum toxin A in the treatment of Meige syndrome [in Chinese]. Acta Acad Med Qingdao Univ 39:394-395.
Wu XL (2003) (OS) Clinical observation of botulinum toxin A for the treatment of Meige syndrome [in Chinese]. Clin Med China 19:138.
Xu JJ, Wang Y, Lu Y, Ye F, Xi GM (2011) (IS) Botulinum toxin A combined with anhydrous ethanol for the treatment of Meige syndrome [in Chinese]. J HBUM 30:194-195.
Zhou SH, Ye F, Fu SZ (2001) (OS) Efficacy of botulinum toxin A in Meige syndrome [in Chinese]. Mod Rehabil 5:82.
Zhou YL, Ding MP (2006) (OS) Botulinum toxin A in Meige syndrome [in Chinese]. J Clin Inter Med 23:558-559.
Secondary Topic Publications
Qu F, Hu JH, Sun YJ, Shan LH, Yu P, Song Y (2003) (OS) Efficacy of botulinum toxin A in 156 patients with hemifacial spasm, blepharospasm and Meige syndrome [in Chinese]. J Apoplexy Nerv Dis 20:68-69.
Wang M, Yu C, Liu RY, Wang JF, Li DF (2002) (OS) Bbotulinum toxin A in 35 cases of hemifacial spasm, blepharospasm and Meige syndrome [in Chinese]. Clin Focus 17:518.
Xu J (2002) (OS) Clincial study on botulinum toxin A therapy of hemifacial spasm, blepharospasm and Meige syndrome [in Chinese]. J Guiyang Med Coll 27:40-41.
Zhu DQ, Zhu T, Yu DC (2012) (IS) Botulinum toxin A combined with GABA drugs in blepharospasm and Meige syndrom [in Chinese]. J Apoplexy Nerv Dis 29:1036-1037.
Cervical Dystonia
Barbosa PM, Rodrigues GR, de Oliveira DS, de Souza CP, Tumas V (2015) (RCT) Comparison between Dysport and Prosigne in the treatment of cervical dystonia Clin Neuropharm 38:221-226.
Chen J, Chen JJ, Lu YQ, Yang K, Liu N (2010) (OS) Clinical study of botulinum toxin A therapy of

spasmodic torticollis [in Chinese]. Chin J Postgrad Med 33:27-29.
Chen LP, Zhou FY, Zhen JZ, Cheng JY (2001) (OS) Botulinum toxin A therapy of 58 cases of cervical dystonia [in Chinese]. Zhejiang Med 23:560-561.
Chen Y, Qiao K, Jiang WX, Jiang YP (2006) (OS) Clinical study of 146 Patients with idiopathic cervical dystonia treated with electromyography guided botulinum toxin A injections [in Chinese]. Chin J Clin Neurosci 14:175-178.
Deng XL, Wang SF (2006) (OS) Botulinum toxin A therapy for 18 cases of cervical dystonia [in Chinese]. Chin J Gen Pract 5:192.
Deng YF, Zeng WS, Wang XH (2009) (OS) Botulinum toxin A therapy for cervical dystonia caused by oral medication [in Chinese]. Chin J Nerv Ment Dis 35:428-429.
Deng ZG, Chen ML, Huang R (2001) (OS) Botulinum toxin A therapy of 86 cases of spasmodic torticollis [in Chinese]. Chin J Phys Med Rehabil 23:382-383.
Fu YG (2001) (OS) Botulinum toxin A therapy for cervical dystonia: a report of 10 cases [in Chinese]. Chin J Nerv Ment Dis 27:226-227.
Ge LT, Wu HJ, Wan Y, Hu Q, Dong DX (2010) (OS) Botulinum toxin A therapy of 79 cases of cervical dystonia [in Chinese]. Neur Injury Funct Reconstr 5:388-389.
Guo JG, Lin HY, Zhu T, Lin J, sun JL, Yao M, Huang B, Zhou XY, Hou J (2012) (OS) Botulinum toxin A combined with radiofrequency thermocoagulation therapy for cervical dystonia [in Chinese]. Chin J Pain Med 18:249-250.
Hu XY, Shao YQ, LV W, Jiang H (2002) (RCT) Comparison of two different concentrations of botulinum toxin A for the treatment of cervical dystonia [in Chinese]. Chin J Phys Med Rehabil 24:721-722.
Huang L, Xiao HQ, Chen HX, Ding XD, Jiang W, Song JH, Hong Y, Zhang GB (2012) (RCT) Efficacy of ultrasound-guided botulinum toxin A injections with orthopaedic joint brace in cervical dystonia [in Chinese]. Chin J Phys Med Rehabil 34:465-467.
Huang L, Chen HX, Ding XD, Xiao HQ, Wang W, Wang H (2015) (RCT) Efficacy analysis of ultrasound-guided local injection of botulinum toxin type A treatment with orthopedic joint brace in patients with cervical dystonia. Eur Rev Med Pharmacol Sci 19:1989-1993.
Jin HW, Zhao ZM, Li J (2002) (OS) Botulinum toxin A therapy for 35 cases of cervical dystonia [in Chinese]. Chin J Pract Intern Med 22:85.
Lei J, Cao LF, Yang ZX, Luo LH (2004) (OS) Clinical analysis of botulinum toxin A therapy of 28 cases of spasmodic torticollis [in Chinese]. Chin J Mod Med 14:115-116.
Liu YH, Sun JL, Chang H (2003) (OS) Clinical study of botulinum toxin A therapy of spasmodic torticollis [in Chinese]. Acta Acad Med Xuzhou 23:147-149.
Lin SZ, Sun AH, Fan JP, Lang JT (2000) (OS) Botulinum toxin A therapy for cervical dystonia [in Chinese]. Chin Arch Otolaryngol Head Neck Surg 7:120-121.
Luo WF, Liu CF, Bao SY, Wu XL, Zhao HQ (2002) (OS) Clinical analysis of electromyography-guided botulinum toxin A therapy of cervical dystonia [in Chinese]. Jiangsu Med J 28:376.
Luo WF, Liu CF, Bao SY, Dai YP, Zhao HQ (2004) (RCT) Comparison of different concentrations of botulinum toxin A for the treatment of cervical dystonia [in Chinese]. J Clin Neurol 17:463-464.
Qiu YH, Wang XY, Zhao QF, Pang WM, Tang YP (2004) (OS) Electromyography-guided botulinum toxin A injections for cervical dystonia: a report of 14 cases [in Chinese]. Chin J Clin Neurosci 12:396-397.
Quagliato EM, Carelli EF, Viana MA (2010) (RCT) A prospective, randomized, double-blind study comparing the efficacy and safety of type a botulinum toxins Botox and Prosigne in the treatment of cervical dystonia. Clin Neuropharm 33:22-26.
Qu F, Hu JH, Liu Y (2002) (OS) Botulinum toxin A therapy of 14 cases of spasmodic torticollis [in Chinese]. Chin J Clin Rehabil 6:3723.

Song CL, Yao JH, Song B (2006) (OS) Botulinum toxin A therapy for cervical dystonia [in Chinese]. J Apoplexy Nerv Dis 23:363.
Song Y, Wang Z, Zhao ZJ, Chen LY, Wei GF (2013) (OS) Efficacy of botulinum toxin A therapy in 48 cases of cervical dystonia [in Chinese]. Shanxi Med J 42:780-781.
Wan XH, Tang XF (1998) (IS) Comparison of Botox and a Chinese botulinum toxin A in cervical dystonia [in Chinese]. Natl Med J China 78:131-134.
Wang HY, Xu LQ, Zhen YY (2010) (OS) EMG-guided botulinum toxin A therapy for 22 cases of cervical dystonia [in Chinese]. J Epileptol Electroneurophysiol (Chin) 19:61-62.
Wang ZY, Liu Y (2006) (OS) Clinical study of botulinum toxin A therapy for cervical dystonia [in Chinese]. Chin J Contemp Neurol Neurosurg 6:147-148.
Wei H, Wang YP, Li LP, Zhang RH, Zhang XJ (2006) (OS) Clinical classification of cervical dystonia and its treatment using Chinese botulinum toxin A [in Chinese]. Chin J Neurol 39:52-54.
Wu CJ, Xue F, Chang WS, Lian YJ, Zheng YK, Xie NC, Zhang L, Chen C (2016) (RCT) Botulinum toxin type A with or without needle electromyographic guidance in patients with cervical dystonia. Springerplus 5:1292-1298.
Yao CY, Wu T, Li M, Hou XD (2011) (OS) Therapeutic efficacy of electromyography-guided botulinum toxin A injections on cervical dystonia [in Chinese]. J Clin Neurol 24:211-212.
Zhang N, Li G, Xiao B, Cai Y, Liu YH (2008) (OS) Therapeutic effects of botulinum toxin A therapy in spasmodic torticollis [in Chinese]. Chin J Rehabil Med 23:629-631.
Zhen DY, Shao YQ, Hu XY (2002) (OS) Clinical analysis of 36 cases with cervical dystonia [in Chinese]. Zhejiang Clin Med 4:820-821.

Craniocervical Dystonia

Deng YF (2000) (OS) Treatment of 196 cases of cranio-cervical dystonia with botulinum toxin A [in Chinese]. J Pract Med 16:996-997.
Dong HJ, Lu ZN, Zeng QX, Yu SZ, Chu H, Yang L (2001) (OS) Botulinum A toxin therapy of cranio-cervical movement disorders [in Chinese]. J Chin Intern Med 18:465-466.
Liang ZH, Song CL, Gao SM, Xu J (2003) (OS) Botulinum A toxin therapy of 100 cases of cranio-cervical dystonia [in Chinese]. Chin J Clin Rehabil 7:4304.
Zhang M, Dai JL, Fang JH (2004) (IS) Clinical observations on botulinum toxin A therapy of cranio-cervical dystonia [in Chinese]. Chin J Neurolmed 13:210-212.
Zhang WX, Wang Y, Liang XL (2003) (OS) Clinical study of botulinum toxin A therapy of cervical dystonia and Meige syndrome [in Chinese]. Chin J Nerv Ment Dis 29:204-205.

Writer' Cramp

Jin HW, Zhou JX (2008) (OS) Botulinum toxin A therapy for writer's cramp: a report of 25 cases [in Chinese]. Clin Med J China 15:568.
Nie JD, Guo ZH, Li SX, Liu GF, Bian YZ (2006) (OS) Botulinum toxin A therapy for writer's cramp [in Chinese]. Chin J Rehabil Med 21:273-274.

Dystonias

Chen L, Xu Z, Shang QZ,Bi XM, Ai QL (2015) (OS) Efficacy and outcome predictors of botulinum toxin A in treatment of focal dystonia [in Chinese]. China Mod Doctor 53:19-22,46.
Hu JY, Wang X, Li K, Hu WB, Han YZ, Cheng N, Yang RM (2010) (OS) Efficacy of botulinum toxin A on patients with serious torsion spasm in hepatolenticular degeneration [in Chinese]. Anhui Med J 31:464-465.
Hu XQ, Li ZJ, Li QH, Guo QX (2003) (OS) Therapeutic effects of botulinum toxin A in the treatment of myospasm [in Chinese]. Chin J Rehabil 18:93-94.
Li YJ, Fu YG (2004) (OS) Botulinum toxin A therapy for dystonia: a report of 409 cases [in Chinese]. Chin J Clin Rehabil 8:2448-2449.
Luo SG, Liang HZ (1999) (OS) Botulinum toxin A therapy for dystonia [in Chinese]. J Guangxi Med

Univ 16:476-477.
Luo SG, Wang J, Cheng DB, Huang J, Ling YX (2007) (OS) Clinical observation of repeated botulinum toxin A injections in focal dystonia [in Chinese]. J Guangxi Med Univ 24:764-765.
Tan Q, Deng YZ, Chen H, Zhen W (2004) (OS) Botulinum toxin A for focal dystonia [in Chinese]. Chin J Clin Rehabil 8:726.
Tang XF, Wan XH, Huang G, Zhang QB, Li T (1999) (OS) The treatment of focal dystonia and muscle spasms with Botox and CBTX-A [in Chinese]. Chin J Neurol 32:135-138.
Wang Q, Rong LQ, Wei XE, Yang S (2008) (OS) Botulinum toxin A in the long-term treatment of focal dystonia [in Chinese]. Chin J Pract Nerv Dis 11:61-63.
Wu YC, Wan H, Huang JW (2000) (OS) Treatment of focal dystonia with botulinum toxin A [in Chinese]. Acta Acad Med Jiangxi 40:25-27.
Yang XL, Ding XG, Weng JY, Ma JH (2002) (OS) Clinical observations on botulinum toxin A therapy of 138 cases of dystonia [in Chinese]. J Xinjiang Med Univ 25:69-70.
Ye XL, Wang FC, Ma XL (2008) (OS) Botulinum A toxin therapy of dystonia [in Chinese]. Chin J Postgrad Med 31:57-58.
Secondary Topic Publications
Lian YJ, Wei HL, Zhang BA, Liu HB, Xu YM, Fang SY, Li ZF, Fang GY, Lu H, Jia YJ, Zhao ZY, Wei JK, Jia YZ, Zhang L, Song B (2009) (OS) Botulinum A toxin therapy of 795 cases of hemifacial spasm and focal dystonia [in Chinese]. J Zhengzhou Univ (Med Sci) 44:440-442.
Hemifacial Spasm
Chen GL, Zhang Q, Hou L, Lin ZC (1999) (OS) Clinical observation on botulinum toxin A treatment of 88 cases of hemifacial spasm [in Chinese]. Chin J Pract Intern Med 19:373-374.
Chen ML, Chai JK, Song HF, Xu MH, Wu YQ, M CH (2007) (OS) Botulinum toxin A for facial hyperkinesias with the botulinum toxin A [in Chinese]. Chin J Aesthetic Med 16:620-622.
Chen YM, Hu CL (2000) (OS) Botulinum toxin A for treatment of hemifacial spasm [in Chinese]. Chongqinq Med J 29:338.
Ding XD, Chen HX, Xiao HQ, Wang W, Wang H, Zhang GB (2015) (RCT) Efficiency of ultrasound and water capsule-guided local injection of botulinum toxin type A treatment on patients with facial spasm. Eur Rev Med Pharmacol Sci 19:1837-1841.
Fu YG (2001) (OS) Treatment of hemifacial spasm and blepharospasm with botulinum toxin A: clinical report of 68 cases [in Chinese]. J Jinan Univ (Med Edition) 22:44-47.
Kong YN (1998) (OS) Botulinum toxin A in 106 cases of hemifacial spasm [in Chinese]. Chin J Clin Neuro Sci 6:219-220.
Li YJ, Fu YG, Zhong ZG, Ding Q, Li ZF, Zhu XZ, Huang Y, Yin WC (2013) (IS) Therapeutic effects of botulinum toxin A at different dilutions in patients with hemifacial spasm: a multicentre, randomised, double-blind and self-crossover controlled trial [in Chinese]. Chin J Contemp Neurol Neurosurg 13:506-511.
Li YJ, Huang Y, Ding Q, Gu ZH, Pan XL (2015) (IS) Evaluation of concentrations of botulinum toxin A for treatment of hemifacial spasm: a randomized double-blind crossover trial. Genet Mol Res 14:1136-1144.
Lian YJ, Wang ZS, Li YQ, Ma KY (2001) (OS) Botulinum toxin A therapy in 20 cases of hemifacial spasm [in Chinese]. J Henan Med Univ 36:208-209.
Lian YJ, Wei HL, Zhang BA, Liu HB, Xu YM, Fang SY, Li ZF, Fang GY, Lu H, Jia YJ, Zhao ZY, Wei JK, Jia YZ, Zhang L, Song B (2009) (OS) Botulinum A toxin therapy of 795 cases of hemifacial spasm and focal dystonia [in Chinese]. J Zhengzhou Univ (Med Sci) 44:440-442.
Liu RG, Fan JB, Wang Y, Ren DY (2002) (OS) Clinical analysis of botulinum toxin A for treatement of hemifacial spasm [in Chinese]. J Clin Stomatol 18:379-380.
Luo WF, Liu CF, Bao SY, Wen ZM, Fu Y (2000) (OS) Botulinum toxin A therapy for hemifacial

spasm and blepharospam [in Chinese]. Acta Acad Med Suzhou 20:237-238.
Luo ZR, Kang QY, Zhou F (2001) (OS) Clinical study of botulinum toxin A therapy for hemifacial spasm and blepharospam [in Chinese]. Chin J Ophthalmol 37:161.
Ma Y, Hu XM, Yuan YJ, Song T, Xu X, Liu Z, Zhang DP, Feng J (2016) (OS) Efficacy of botulinum toxin A in the treatment of hemifacial spasm [in Chinese]. Pract Pharm Clin Rem 19:584-587.
Nie JT, Liu SX, Ha ZY, Yu ZY (1997) (OS) Botulinum toxin A in 108 cases of hemifacial spasm and blepharospasm [in Chinese]. J Apoplexy Nerv Dis 14:43-45.
Peng B, Dong HJ, Chu H, Zhang SQ, Lu ZN (2015) (RCT) Clinical and electrophysiological studies of botulinum toxin A for treatment of hemifacial spasm complicated with auricular symptoms. Int J Clin Exp Med 8:9772-9778.
Peng B, Zhang SQ, Dong HJ, Luo Y, Lu ZN (2017) (OS) The impact of botulinum toxin on anxiety and depression in patients with hemifacial spasm and blepharospasm [in Chinese]. Chin J Nerv Ment Dis 43:603-607.
Peng B, Zhang SQ, Dong HJ, Luo Y, Lu ZN (2017) (OS) Clinical and therapeutic features of patients with hemifacial spasm and benign essential blepharospasm [in Chinese]. Nerv Dis Ment Health 17:173-176.
Qu F, Hu JH, Sun YJ, Shan LH, Yu P, Song Y (2003) (OS) Efficacy of botulinum toxin A in 156 patients with hemifacial spasm, blepharospasm and Meige syndrome [in Chinese]. J Apoplexy Nerv Dis 20:68-69.
Rong LQ, Wang Q, Yang S (2000) (OS) Botulinum toxin A in hemifacial spasm and blepharospasm [in Chinese]. Acta Acad Med Xuzhou 20:220-222.
Shi XL, Wang CQ, Meng Y, Fu J (2014) (OS) Long-term therapeutic efficacy of botulinum toxin A in treatment of hemifacial spasm [in Chinese]. Anhui Med J 35:884-886.
Sun SF, Meng FQ, Ma YM, Fu YC (2003) (OS) Botulinum toxin therapy of hemifacial spasm [in Chinese].Chin J Coal Industry Med 6:343-344.
Wang GD, Xu ZX (2008) (OS) Clinical observation on botulinum toxin A treatment of 59 cases of hemifacial spasm and blepharospasm [in Chinese]. Hainan Med J 19:27-28.
Wang J (2009) (OS) Botulinum toxin A for treatment of hemifacial spasm [in Chinese]. Chin J Pract Nerv Dis 12:56-57,45.
Wang L, Hu XY, Dong HJ, Wang WZ, Huang Y, Jin LJ, Luo YM, Zhang WX, Lian YJ, Liang ZH, Shang HF, Feng YB, Wu YW, Chen J, Luo WF, Wan XH (2014) (OS) Clinical features and treatment status of hemifacial spasm in China. Chin Med J 127:845-849.
Wang M, Yu C, Liu RY, Wang JF, Li DF (2002) (OS) Bbotulinum toxin A in 35 cases of hemifacial spasm, blepharospasm and Meige syndrome [in Chinese]. Clin Focus 17:518.
Wang WZ, Zhang LM, Yan XB, Chen GR, Peng JC, Zhao YJ (1997) (OS) Botulinum toxin A in the treatment of hemifacial spasm and blepharospasm [in Chinese]. Chin J Pract Intern Med 17:674-675.
Wang WZ, Zhao ZX, Xia B, Huang J, Zhao Y, Huang LQ, Zhou H, He B (2007) (OS) Treatment of facial spasms with botulinum toxin A: a report of 54 cases [in Chinese]. Acad J Sec Mil Med Univ 28:1389-1390.54
Xiao LB, Pan YG, Zhang XL, Hu Y, Cai L, Nie ZY, Pan LZ, Li B, He YJ, Jin LJ (2016) (OS) Facial asymmetry in patients with hemifacial spasm before and after botulinum toxin A therapy. Neuro Sci 37:1807-1813.
Xu GF, Bao H, Wang XL, Wan CH, He LQ, Liu YJ (2008) (OS) Clinical study of botulinum toxin A treatment of hemifacial spasm and blepharospasm [in Chinese]. China Mod Doctor 46:174-176
Xu J (2002) (OS) Clincial study on botulinum toxin A therapy of hemifacial spasm, blepharospasm and Meige syndrome [in Chinese]. J Guiyang Med Coll 27:40-41.
Xu YP, Shen J, Zhu QB, Gu J, Lin SZ, Fan JP (2013) (OS) Efficacy of botulinum toxin A for treatment of unilateral spasms of the eyelid and its prognosis. Eur Rev Med Pharmacol Sci 17:2974-2979.

Yin LX, Zhang WZ (2003) (OS) Clinical observation of botulinum toxin A treatment of 65 cases of hemifacial spasm [in Chinese]. J Xinjiang Med Univ 26:189.
Zhang R (2002) (OS) Botulinum toxin A in hemifacial spasm: a report of 80 cases [in Chinese]. J Xinjiang Med Univ 25:65.
Zhao XM, Ma L (2006) (IS) Therapeutic efficacy of botulinum toxin A in hemifacial spasm and blepharospasm [in Chinese]. Zhejiang Clin Med 8:1035-1036.
Secondary Topic Publication
Chen GZ, Gao C (2001) (OS) Treatment of blepharospasm and hemifacial spasm with botulinum toxin A [in Chinese]. Chin J Optom Ophthalmol 3:182-183.
Chen XH, Zhuang ZH (2006) (OS) Effects of botulinum toxin A on idiopathic blepharospasm and facial spasm [in Chinese]. Chin J Pract Nerv Dis 9:15-16.
Fan YH (2010) (OS) Clinical observations on botulinum toxin A therapy for blepharospam and hemifacial spasm [in Chinese]. Chin J Aesthetic Med 19:759.
Kang XL, Gan CZ (2008) (OS) Clinical study of botulinum toxin A treatment of blepharospasm and hemifacial spasm [in Chinese]. Chin J Pract Nerv Dis 11:15-16.
Lan ZH, Zhang LZ (2001) (OS) Clinical observations on botulinum toxin A therapy of 364 cases of blepharospasm and hemifacial spasm [in Chinese]. Chin J Strabismus Pediatr Ophthalmol 9:158-159.
Li HM (2001) (OS) Treatment of blepharospasm and hemifacial spasm with botulinum toxin A [in Chinese]. J Pract Aesth Plasti Surg 12:194.
Rieder CR, Schestatsky P, Socal MP, Monte TL, Fricke D, Costa J, Picon PD (2007) (IS) A double-blind, randomised, crossover study of prosigne versus botox in patients with blepharospasm and hemifacial spasm. Clin Neuropharmacol 30:39-42.
Tong Y, Xu TH, Fan YX (1999) (OS) Botulinum toxin A for blepharospasm and hemifacial spasm [in Chinese]. J Clin Ophthalmol 7:330-331.
Wang J, Fei XQ, Zhao LL, Lu W (2004) (OS) Treatment of blepharospasm and hemifacial spasm with botulinum toxin A: clinical analysis of 657 cases [in Chinese]. Chin J Strabismus Pediatr Ophthalmol 12:37-38.
Wang JL, Zhang X (2006) (OS) Efficacy of repeated botulinum toxin A injections on blepharospasm and hemifacial spasm [in Chinese]. J Clin Otorhinolaryngol China 20:292-294.
Wu CJ, Shen JH, Chen Y, Lian YJ (2011) (IS) Comparison of two different formulations of botulinum toxin A for the treatment of blepharospasm and hemifacial spasm. Turk Neurosurg 21:625-629.
Xian WG (2003) (OS) Treatment of idiopathic blepharospasm and hemifacial spasm with botulinum toxin A [in Chinese]. China Trop Med 3:47-48.
Xiang YH, Fu Y (2008) (OS) The effect of botulinum toxin A in treatment of blepharospasm and hemifacial spasm [in Chinese]. J Clin Clin Exp Med 7:98-99.
Xu TH, Tong Y, Fan YX (2001) (OS) Clinical study on botulinum toxin A therapy of blepharospasm and Meige syndrome [in Chinese]. J Clin Ophthalmol 19:415-416.
Yin XX, Yang XG, Zhu SL, Chu MF (2005) (OS) The clinical effects of botulinum toxin A therapy of blepharospasm and hemifacial spasm [in Chinese]. Chin J Pract Ophthalmol 23:1128-1159.
Zeng L (2012) (OS) Efficacy of botuliunum toxin A in the treatment of blepharospasm and hemifacial spasm [in Chinese]. Chin J Strabismus Pediatr Ophthalmol 20:151-153,156.
Zhao J (2002) (OS) Treatment of blepharospasm and hemifacial spasm with botulinum toxin A [in Chinese]. Ophthalmol China 11:227-228.
Zhao SY (2009) (OS) Non-surgical treatment of 150 cases of blepharospasm and hemifacial spasm [in Chinese]. Chin J Optom Ophthalmol 11:232-233.
Tics
Wang L, Wan XH, Li LB (2010) (OS) Botulinum toxin A therapy of tic disorder [in Chinese]. Chin J

Neurol 43: 65-68.

Spasmodic Dysphonia

Hu XY, fan ZG, Jiang H, Zhang SZ, Shao YQ (2002) (RCT) Botulinum toxin A therapy of spasmodic dysphonia [in Chinese]. Chin J Otorhinolaryngol 37:466-467.
Wang JJ, Liang YF, Wei CS, Zhang Y, Jiang JQ (2012) (OS) Botulinum toxin A therapy with laryngoscopy for adductor spasmodic dysphonia [in Chinese]. Chin J Otorhinolaryngol Head Neck Surg 47:601-602.
Xu W, Han DM, Hou LZ, Zhang L, Gao YH, Ye JY, Wang J (2005) (OS) Patterns of spasmodic dysphonia and botulinum toxin therapy [in Chinese]. Chin J Otorhinolaryngol Head Neck Surg 40:253-257.

Spasticity

Ao LJ, Qian QH, Zhen L, Tang M, Wang WL, Xu JH (2003) (OS) Botulinum toxin A for treatment of spasticity after spinal cord injury [in Chinese]. Chin J Rehabil Med 18:94-96.
Chen FJ, Chen ZY, Liang XZ, lin HF (2003) (RCT) Botulinum toxin A for functional recover in severe post-stroke limb spasticity [in Chinese]. Chin J Clin Rehabil 7:3478-3479.
Chen YX, Ni YY, Qiu CY, Zhang Y, Li XD, Liu YY, Chen X, Lai XX, Feng CR, Zhang TT (2011) (IS) Repeated injections of botulinum toxin A for leg spasticity after stroke [in Chinese]. Chin J Rehabil Theory Pract 17:449-451.
Cui LH, Lu HT (2006) (OS) Efficacy of botulinum toxin A on post-stroke arm spasticity with different functional status [in Chinese]. Chin J Rehabil Theory Pract 12:1088-1089.
Cui LH, Zhang T (2006) (RCT) Botulinum toxin A therapy for post-stroke arm spasticity [in Chinese]. Chin J Neurol 39:463-466.
Ding XD, Chen HX, Wang W, Wang H, Huang L (2014) (RCT) Efficacy of botulinum toxin A combined with ankle·foot orthosis on patients with post-stroke leg spasticity [in Chinese]. Chin J Phys Med Rehabil 36:349-352.
Ding XD, Zhang GB, Chen HX, Wang W, Song JH, Fu DG (2015) (RCT) Color Doppler ultrasound-guided botulinum toxin type A injection combined with an ankle foot brace for treating lower limb spasticity after a stroke. Eur Rev Med Pharmacol Sci 19:406-411.
Ding XD, Huang L, Wang QS, Liu YP, Zhong J, Chen HX (2017) (RCT) Botulinum toxin A combined with spasmodic muscle therapeutic instrument on lower limb spasticity in patients with stroke. Exp Ther Med 13:3319-3326.
Dou ZL, Tao QF, Qiu WH, Hu XQ (2001) (OS) Botulinum toxin A for leg spasticity after brain damage [in Chinese]. Chin J Phys Med Rehabil 23:325-327.
Dou ZL, Tao QF, Hu XQ, Qiu WH (2003) (OS) Botulinum toxin A in the treatment of leg spasticity after stroke and head injury [in Chinese]. Chin Pharmacol Bull 19:197-200.
Dou ZL, Ou HN, Shen JH, Yuan XF, Lan Y (2008) (OS) Efficacy of ultrasound-guided botulinum toxin A injections in treating post-stroke spasticity: report of 5 cases [in Chinese]. Chin J Rehabil Med 23:219-221.
Guarany FC, Picon PD, Guarany NR, dos Santos AC, Chiella BP, Barone CR, Fendt LC, Schestatsky P (2013) (IS) A double-blind, randomised, crossover trial of two botulinum toxin type a in patients with spasticity. PLoS One 8:e56479.
Hu Q, Ge LT, Jiang HW, Wu HJ (2014) (IS) Efficacy of botulinum toxin A combined with rehabilitation on motor function in post-stroke patients [in Chinese]. Chin J Rehabil 29:107-108.
Lai XX, Chen YX, Liu CR, Zhang XF, Zeng PJ, Zhang YH (2013) (RCT) Efficacy of botulinum toxin A on leg spasticity after stroke [in Chinese]. Chin J Rehabil Theory Pract 19:469-471.
Lan Y, Dou ZL, Hu XQ, Xu GQ, Qiu WH (2007) (RCT) Clinical study of botulinum toxin A in the treatment of arm spasticity of stroke patients [in Chinese]. Chin J Phys Med Rehabil 29:754-757.
Lan Y, Xu GQ, Hu XQ, Li K, Huang DF (2007) (RCT) Effects of botulinum toxin A and

comprehensive anti-spasticity treatment on quality of life of stroke survivors [in Chinese]. Chin J Rehabil Med 22:912-914.
Li J, Zhang R, Cui BL, Zhang YX, Bai GT, Gao SS, Li WJ (2018) (RCT) Therapeutic efficacy and safety of various botulinum toxin A doses and concentrations in spastic foot after stroke: a randomized controlled trial. Neural Regen Res 12:1451-1457.
Li YB, Feng HX, Bai JJ, Su ML, Han LY, Bao AQ, Liang XB, Qiao XH (2017)(RCT) Efficacy of botulinum toxin A combined with intensive physical therapy on walking ability in patients with post-stroke leg spasticity [in Chinese]. Chin J Pract Nerv Dis 20:5-9.
Li YH, Wang FY, Sun YH, Yang XF, XU J (2009) (OS) Botulinum toxin A with colour Doppler ultrasound guidance for post-stroke spasticity [in Chinese]. Chin J Rehabil Theory Pract 15:578-579.
Luo SG, Wang J, Wu XP, Cheng DB, Long YB (2009) (RCT) Botulinum toxin A combined with rehabilitation training for post-stroke spastic leg paralysis [in Chinese]. Chin J Rehabil Med 24:817-820.
Meng L, Lin GT, Tang XZ (2008) (RCT) Efficacy of botulinum toxin A on arm spasticity after stroke [in Chinese]. Chin J Neurolmed 7:740-742.
Ou HN, Lu WY, Zhao SH, Liu YF, Nong WH (2014) (RCT) Shujin Huoluo Lotion and botulinum Toxin A in the treatment of post-stroke arm spasticity [in Chinese]. Chin Med Herald 11:98-102.
Ou HN, Huang B, Zhou KX, Huang ZQ, Deng WX, Lu WY (2015) (RCT) Comparison of palpation, ultrasound and electromyography guidance for botulinum toxin A injections for equinovarus in stroke [in Chinese]. Chin J Rehabil Theory Pract 21:706-712.
Qi JT, Yu D, Gao J, Yang JS, Li JQ (2012) (OS) Botulinum toxin A for treatment of severe arm spasticity in patients with reduced consciousness [in Chinese]. Chin J Rehabil Med 27:240-243.
Ren LJ, Han MF, Bai RT, Feng HY, Shao YF (2008) (RCT) Botulinum toxin A in treatment of spastic foot drop and inversion after stroke [in Chinese]. Chin J Pract Nerv Dis 11:11-13.
Shen JH, Ou HN, Shen Q, Cui JC, Chen HX (2008) (OS) Ultrasound guided botulinum toxin injections for treatment of limb spasticity after stroke [in Chinese]. J Guangdong Coll Pharm 24:518-520.
Song T, Long LP, Li HP (2013) (IS) Efficacy of botulinum toxin A on tibial nerve F-wave and walking ability of stroke patients [in Chinese]. Chin J Phys Med Rehabil 35:119-122.
Wang CX, Sun SC, Wu CJ (2007) (OS) Botulinum toxin A for post-stroke limb spasticity [in Chinese]. Med J Qilu 22:386-387.
Wang RC (2013) (RCT) Botulinum toxin A combined with rehabilitation for post-stroke arm spasticity [in Chinese]. Chin J Gerontol 33:5354-5355.
Xia Q (2017) (CS) Botulinum toxin A injections with ultrasound guidance in post-stroke spasticity [in Chinese]. Anhui Med J 38:1507-1508.
Xing ST, Wang D, Wen XH, Wu ZQ, Sun Q, Zhang DW, Cheng Y, Yan D, Yu F (2010) (IS) Clinical research of electroacupuncture combined with acupoint-injection of botulinum toxin A in treating spasticity after spinal cord injury [in Chinese]. China J Orthop Traumatol 23:350-353.
Xu JX, Ma C, Wu SL, Ou LM, Yan TB (2004) (OS) Efficacy of botulinumtoxin A in flexor arm spasticity after stroke [in Chinese]. Chin J Clin Rehabil 8:12-13.
Yang XY, Xu GX, Mao YJ, Gu SX, Wang J, Jin TJ, Li JA (2005) (IS) Efficacy of botulinum toxin A on spasticity [in Chinese]. Chin J Rehabil Med 20:675-679.
Yang YM, Liang Q, Wan XH, Wang L, Chen SL, Wu Q, Zhang XP, Ding SY, Shang HF, Hu XY, Lu JH, Tao EX, Nie ZY, Pan XD, Tang RH, Zhang BR, Chen J, Tan HY, Dong HJ, Li JA, Luo WF, Yao C (2018) (RCT) Safety and efficacy of botulinum toxin A made in China for treatment of post-stroke arm spasticity：a randomised, double-blind controlled trial [in Chinese]. Chin J Neurol 51:355-363.
Yu M, Huang G, Fu M, Liu YQ (2004) (OS) Botulinum toxin A injections with electromyography guidance in spasticity after stroke [in Chinese]. Chin J Rehabil Theory Pract 10:742-743.

Zhang M, Li ZX, Dai JL, Fang JH, Yu CQ (2001) (OS) Efficacy of botulinum toxin A in post-stroke spasticity [in Chinese]. J Clin Neurol 14:212-214.
Zhang MB (2010) (RCT) Efficacy of botulinum toxin A combined with acupuncture for post-stroke arm spasticity [in Chinese]. Chin J Misdiagn 10:1286-1287.
Zhang XL, Yu FC, Zheng XQ, Peng DS (2006) (IS) Efficacy of botulinum toxin A on post-stroke spasticity [in Chinese]. Chin J Rehabil 21:32-34.
Zhao YR, Li CC, Cui QF (2005) (OS) Botulinum toxin A for improving spastic gait in patients with cerebral apoplexy [in Chinese]. Chin J Clin Rehabil 9:4-5.
Zhou SH (2003) (OS) Efficacy of botulinum toxin A combined with medium frequency electric stimulation in post-stroke spasticity [in Chinese]. Chin J Phys Med Rehabil 25:307.

Cerebral Palsy

Ao LJ, Qian JH, Wang WL, Zheng L, Tang M, Yao LQ, Li YM (2005) (OS) Efficacy of electromyography-free injections of botulinum toxin A in the treatment of children with spastic cerebral palsy [in Chinese]. Chin J Rehabil Med 20:915-917.
Chen Q, Xu HQ, Jin SF, Su KY, Huang ZH, Zou SH (2012) (RCT) Efficacy of botulinum toxin A and herbal bath on spasticity of children with cerebral palsy [in Chinese]. Chin J Rehabil Theory Pract 18:916-918.
Dong XL, Kong M, Yu ZH, Wu RM, Liu W (2008) (RCT) Efficacy of botulinum toxin A in cerebral palsy [in Chinese]. West China Med J 23:844-845.
Du FZ, Lu SL, Wang HY, Xi CP, Ding JF, Jiang Y, An CW, Wei YX (2003) (RCT) Botulinum toxin A in treatment of spastic cerebral palsy [in Chinese]. Chin J Rehabil Theory Pract 9:328-329.
Duan FH, Zhang ZX (2018) (RCT) Efficacy of botulinum toxin therapy of pediatric spastic cerebral palsy and its effect on prognosis [in Chinese]. Chin J Prim Med Pharm 25:613-616.
Gao BQ, Yang WL, Deng X, Zhao ZG, Wang YJ, Li FW (2001) (OS) Efficacy of botulinum toxin A in leg spasticity of children with cerebral palsy [in Chinese]. Chin J Nerv Ment Dis 27:448-449.
Gao BQ, Yang WL, Zhu BJ, Deng X, Wang YJ, Deng YX, Tian ZX, Wang GF (2003) (IS) Different doses of botulinum toxin A for treatment of spastic cerebral palsy [in Chinese]. J Capital Univ Med Sci 24:73-75.
Gao BQ, Yang WL, Wang YJ, Tian CX, Deng YX (2005) (OS) Botulinum toxin A in the treatment of spastic cerebral palsy [in Chinese]. Chin J Neuromed 4:488-490.
Gao BQ, Zhao XQ, Xie JL, Deng X, Li FW (2006) (RCT) Efficacy of rehabilitation and botulinum toxin A on spastic cerebral palsy [in Chinese]. Chin J Rehabil Theory Pract 12:101-102.
Huang Y, Li RH, Que LS (2007) (RCT) Efficacy of botulinum toxin A on spastic cerebral palsy [in Chinese]. China Trop Med 7:729-730.
Liang S, Liu HT, Ke HC, Zhao Y, Li XB, Gao CX (2003) (RCT) Botulinum toxin, Ueda physiotherapy and traditional Chinese medicine in the treatment of cerebral palsy [in Chinese]. Chin J Phys Med Rehabil 25:610-613.
Lin B, Xie HX, Liu N, wang JL, Sun MF (2013) (RCT) Botulinum toxin A for treatment of triceps surae spasticity in children with cerebral palsy [in Chinese]. Chin J Rehabil 28:370-371.
Liu JJ, Ji SR, Hu YY, Li YC, Wu WH, Lu HB, Zhang Y (2003) (IS) Efficacy of botulinum toxin A in spastic cerebral palsy [in Chinese]. Chin J Phys Med Rehabil 25:669-671.
Liu JJ, Ji SR, Hu YY, Li YC, Wu WH, Lu HB, Zhang Y (2006) (OS) Optimal dose of botulinum toxin A in the treatment of spastic cerebral palsy [in Chinese]. Chin J Rehabil Theory Pract 12:831-832.
Liu JJ, Ji SR, Hu YY, Wu WH, Li CY, Lu HB, Zhang Y (2008) (OS) Optimal dose of botulinum toxin A to release spasticity of triceps surae in children with cerebral palsy [in Chinese]. Chin J Rehabil Theory Pract 14:1173-1176.
Liu JJ, Ji SR, Hu YY, Wu WH, Li YC, Lu HB, Zhang Y (2008) (OS) Releasing triceps surae spasticity in children with spastic cerebral palsy by botulinum toxin A doses calculated according to a special

formula [in Chinese]. Chin J Rehabil Theory Pract14:956-957.
Liu JJ, Ji SR, Hu YY, Wu WH, Li YC, Lu HB, Zhang Y (2008) (OS) Suitable botulinum toxin A doses and correlation factors for treatment of spastic cerebral palsy [in Chinese]. Chin J Rehabil Theory Pract 14:358-360.
Liu JJ, Ji SR, Wu WH, Zhang Y, Zeng FY, Li NL (2013) (RCT) Botulinum toxin-A with and without rehabilitation for the treatment of spastic cerebral palsy. J Int Med Res 41:636-641.
Liu JJ, Ji SR, Wu WH, Zhang Y, Zeng FY, Li NL (2013) (IS) Efficacy of botulinum toxin A injections into the spastic iliopsoas in children with cerebral palsy [in Chinese]. Chin J Rehabil Theory Pract 19:956-959.
Liu JJ, Ji SR, Wu WH, Zhang Y, Zeng FY, Li NL (2014) (RCT) Botulinum toxin A into the spastic iliopsoas in children with cerebral palsy. Eur Rev Med Pharmacol Sci 18:3223-3228.
Liu JJ, Qi J, Zhang Y, Zeng FY, Li NL, Xi BY, Wu WH (2017) (OS) Efficacy of botulinum toxin A on spastic cerebral palsy: two years follow-up [in Chinese]. Chin J Rehabil Theory Pract 23:816-819.
Ma CY, Wu L, Geng XJ (2006) (OS) Effect of low doses of botulinum toxin A in spastic cerebral palsy [in Chinese]. J Med Forum 27:23-25.
Niu GH, Zhang XL, Zhu DN, Cai ZJ, Li SS, Zhang W (2014) (RCT) Efficacy of different doses of botulinum toxin A on tiptoe deformation in children with cerebral palsy [in Chinese]. Chin J Contemp Pediatr 16:720-724.
Peng GL, Cai SY (2011) (RCT) Efficacy of botulinum toxin A with electromyographic guidance in spastic cerebral palsy [in Chinese]. Clin J Pediatr 29:1084-1086.
Peng GL, Cai SY (2011) (IS) Efficacy of different doses of botulinum toxin A in the treatment of spastic cerebral palsy [in Chinese]. Chin J Neurolmed 10:630-632.
Ran X, Wang HY, Xu QL (2005) (RCT) Management of spastic paediatric cerebral palsy using botulinum toxin A and/or physiotherapy [in Chinese]. Chongqing Med J 34:674-675.
Shao YJ, Wu GH, Zeng KH (2013) (OS) Botulinum toxin type A guided by peripheral nerve stimulation for spastic cerebral palsy [in Chinese]. Chin J Rehabil Theory Pract 19:883-884.
Wang Q, Luo R, Lv X, Li N, Ma D, Mu DZ, Yu T (2012) (IS) Combining serial casting with botulinum toxin A in the treatment of spastic equinus in children with cerebral palsy [in Chinese]. Chin J Phys Med Rehabil 34:760-763.
Wang YH (2008) (OS) Botulinum toxin A injections guided by electric stimulation combined with rehabilitation in the treatment of spastic cerebral palsy [in Chinese]. J Appl Clin Pediatr 23:454-456.
Wang YJ, Gao BQ, Yang WL, Han YJ (2004) (OS) Botulinum toxin A in the treatment of spastic cerebral palsy [in Chinese]. Chin J Pract Pediatr 19:546-548.
Wang YJ, Gao BQ, Yang WL (2005) (OS) Botulinum toxin A for treatment of spastic cerebral palsy [in Chinese]. J Appl Clin Pediatr 20:799-800.
Xu KS, Yan TB, Mai JN (2006) (RCT) Efficacy of botulinum toxin therapy guided by electric stimulation on spasticity in ankle plantar flexor of children with cerebral palsy: a randomised trial [in Chinese]. Chin J Pediatr 44:913-917.
Xu L, Wang JW, Yu HF, Yang YL, Li XX, Sang L, Huang Y, Zhao XL (2007) (IS) Botulinum toxin A combined with rehabilitation training for spastic diplegia in juvenile cerebral palsy [in Chinese]. Chin J Phys Med Rehabil 29:121-124.
Xu L, Yang YL, Yu HF, Ma HX, Huang Y, Sang L, Li HY (2008) (RCT) Botulinum toxin A in the leg treatment of spastic hemiplegic cerebraI palsy in children [in Chinese]. CJCHC 16:704-706.
Xu QL, Zou L, Yi GX, wang MS (2004) (RCT) Botulinum toxin A in adjunctive treatment of children with cerebral palsy [in Chinese]. Chongqing Med J 33:869-870.
Yang HF, Zuo YX, Li AX, Li JM, Li QH, Zheng HC, Zhao YX (2012) (RCT) Botulinum toxin A therapy of cerebral palsy [in Chinese]. Hebei Med J 34:359-360.
Yu Y, Han FY, Cui QB (2005) (OS) Botulinum toxin A for treatment of arm spasticity in children

with cerebral palsy：quantification of drug administration and efficacy [in Chinese]. Chin J Clin Rehabil 9:172-175.
Zhao CM, Yu RG, Liao W, Zhang YP, Ai YP, Xi M, Yao L, Liu L (2002) (OS) Botulinum toxin A in the treatment of leg spasticity in cerebral palsy [in Chinese]. Chin J Clin Rehabil 6:2853-2854.

Strabismus

Chen J, Deng D, Zhong H, Lin X, Kang Y, Wu H, Yan J, Mai G (2013) (RCT) Botulinum toxin injections combined with or without sodium hyaluronate in the absence of electromyography for the treatment of infantile esotropia: a pilot study. Eye 27:382-386.
Liu G, Wu X, Kong J (2006) (OS) Effect of botulinum toxin A on strabismus and diplopia in the patients with Graves' ophthalmopathy [in Chinese]. Rec Adv Ophthalmol 26:296-298.
Liu XH, Pei CG, Shao Y, Miao CY, Huang XB (2011) (OS) Application of botulinum toxin A guided by high-frequency ultrasound in the treatment of restrictive strabismus in thyroid associated ophthalmopathy [in Chinese]. Int J Ophthalmol 11:1070-1071.
Li Y, Wu X (2008) (IS) Botulinum toxin A treatment of children with intermittent exotropia [in Chinese]. Chin J Ophthalmol 44:967-971.
Li Y, Wu X, Wang JH (2008) (OS) Short-term results of botulinum toxin A treatment of childhood intermittent exotropia [in Chinese]. Ophthalmol China 17:126-129.
Shao XX, Si MY, Fan KS (2017) (OS) Treatment of paralytic esotropia by transposition of superior rectus muscle combined with modified muscle connection surgery and botox injection [in Chinese]. Chin J Strabismus Pediatr Ophthalmol 25:11-13.
Shao Y, Hu PH, Zou J, Zhang Y, Liu XH, Pei CG (2015) (OS) Efficacy of muscle belly orbit-ball silicon tube connection and botulinum toxin type A extraocular muscle injection for paralytic strabismus [in Chinese]. Rec Adv Ophthalmol 35:832-835.
Sun SY, Wu GY, Su YM (2002) (OS) Botulinum toxin A in the treatment of paralytic strabismus [in Chinese]. Rec Adv Ophthalmol 22:250.
Wan XM, Chu RX, Gong HQ (2011) (OS) Minimally invasive botulinum toxin type A injection from the ocular surface to extraocular muscles. Int J Ophthalmol (Engl) 4:179-181.
Wang X, Wu X (2009) (IS) Short-term outcome of treatment of acquired concomitant esotropia with botulinum toxin A [in Chinese]. Chin J Strabismus Pediatr Ophthalmol 17:1-5.
Wang X, Wu X (2010) (OS) The role of botulinum toxin for acute-onset concomitant esotropia：a pilot study [in Chinese]. Ophthalmol China 19:110-112.
Wen Y, Wan LQ, Wan XM, Gong HQ (2012) (OS) Minimal invasive injection of botulinum toxin type A for strabismus [in Chinese]. Rec Adv Ophthalmol 32:379-381.
Wu X (2002) (OS) Botulinum toxin A in the treatment of sixth cranial nerve palsy [in Chinese]. Chin J Ophthalmol 38:457-461.
Wu X, Lin N, Ai LK, Wang JH, Yan LJ (2006) (OS) Application of botulinum toxin A for treatment of restrictive strabismus in thyroid associated ophthalmopathy [in Chinese]. Chin J Ophthalmol 42:1063-1067.
Wu X, Wang KM, Wang JH (1999) (OS) Botulinum toxin combined with surgery in the treatment of sixth nerve palsy in the late stage [in Chinese]. Chin J Strabismus Pediatr Ophthalmol 7:127-130.
Zeng L, Hao GS, Lu W, Li YR, Shui D (2011) (OS) Subtenon injection of botulinum toxin A for treatment of paralytic strabismus without electromyographic guidance [in Chinese]. Chin J Strabismus Pediatr Ophthalmol 19:110-113.
Zhong HH, Chen JC, Mai GH, Deng HW, Deng DM (2016) (OS) Study of botulinum toxin A gel for treatment of infantile esotropia [in Chinese]. Chin J Pract Ophthalmol 34:868-871.
Zhong HH, Chen JC, Deng HW, Liu CM (2016) (OS) Botulinum toxin A injection with Jensen procedure for the treatment of acquired lateral rectus muscle palsy [in Chinese]. Chin J Strabismus Pediatr Ophthalmol 24:18-24.

Secondary Topic Publications
Lou XB, Dai S, Li YZ, Liu XY (2005) (OS) Clinical use of botulinum toxin A in blepharospasm and strabismus [in Chinese]. Chin J Strabismus Pediatr Ophthalmol 13:173-174.
Bladder Dysfunctions
Chen GQ, Liao LM (2011) (OS) Injections of botulinum toxin A into the detrusor to treat neurogenic detrusor overactivity secondary to spinal cord injury. Int Urol Nephrol 43:655-662.
Fu G, Wu J, Cong HL, Zha LH, Li D, Ju YH, Chen GQ, Xion ZS, Liao LM (2015) (RCT) Efficacy of botulinum toxin A to treat neurogenic incontinence in patients with spinal cord injury: comparison of two doses [in Chinese]. Natl Med J China 95:3920-3923.
Gao Y, Liao LM (2015) (OS) Intravesical injection of botulinum toxin A for treatment of interstitial cystitis/bladder pain syndrome: 10 years of experience at a single center in China. Int Urogynecol J 26:1021-1026.
Gomes CM, Castro Filho JE, Rejowski RF, Trigo-Rocha FE, Bruschini H, Barros Filho TE, Srougi M (2010) (IS) Experience with different botulinum toxins for the treatment of refractory neurogenic detrusor overactivity. Int Braz J Urol 36:66-74.
Jia C, Liao LM, Chen G, Sui Y (2013) (OS) Detrusor botulinum toxin A injection significantly decreased urinary tract infection in patients with traumatic spinal cord injury. Spinal Cord 51:487-490.
Li D, Liao LM, Song ZS, Fu G, Ju YH, Wu J, Han CS, Shi WB, Huang Y (2005) (OS) Repeated injections of botulinum toxin A into the detrusor to treat detrusor hyperreflexia and neurogenic incontinence in spinal cord injury patients: report of 6 cases [in Chinese]. Chin J Rehabil Theory Pract 11:897-898.
Li JZ, Liu JL, Guo Y, Sha J, Li Q (2014) (RCT) Efficacy of botulinum toxin A in the treatment of female overactive bladder [in Chinese]. Prog Mod Biomed 14:163-165.
Meng ZX, Wang T, Yin ZL, Wang JB (2015) (RCT) Electroacupuncture combined with transperineal injection of botulinum toxin A for neurogenic bladder after spinal cord injury [in Chinese]. Chin Acupunct Moxib 35:17-20.
Temporomandibular Joint Disorders
Fu KY, Chen HM, Sun ZP, Zhang ZK, Ma XC (2010) (OS) Long-term efficacy of botulinum toxin type A for the treatment of habitual dislocation of the temporomandibular joint. Br J Oral Maxillofac Surg 48:281-284.
Fu KY, Zhang W, Cao Y, Kang YF, Xie QF (2012) (OS) Classification and clinical characteristics of masticatory spasms [in Chinese]. Chin J Stomatol 47:423-426.
Jiang MY, You QL (2016) (RCT) Efficacy of semiconductor laser therapy combined with botulinum toxin A injections in the treatment of temporomandibular disorders [in Chinese]. Shanghai J Stomatol 25:758-761.
Gastroparesis
Yin GJ, Tan W, Hu DM (2016) (CS) Endoscopic ultrasonography-guided intrapyloric injection of botulinum toxin to treat diabetic gastroparesis. Dig Endosc 28:755-759.
Achalasia
He X, Tang XD, Fan H, Zhou Y (2001) (OS) Oesophagus kinematics before and after treatment of cardia achalasia with botulinum toxin A [in Chinese]. Chin J Gastroenterol Hepatol 10:158-160.
Yu ZH, Tao JS, Zhang KR (2012) (OS) Endoscopic botulinum toxin A injections for 26 cases of cardia achalasia [in Chinese]. Med J NDFNC 33:166-167.
Oesophagus Strictures
Wen J, Lu ZS, Linghu EQ, Yang YS, Yang J, Wang SF, Yan B, Song J, Zhou XD, Wang XD, Meng K, Dou Y, Liu QS (2016) (RCT) Prevention of esophageal strictures after endoscopic submucosal dissection with the injection of botulinum toxin type A. Gastrointest Endosc 84:606-613.

Dysphagia
Yue SW, Huai J, Guan JW, Yu RX, Liu L (2017) (CS) Botulinum toxin A injections into the cricopharyngeal muscle to treat dysphagia following brain stem injury. Combination with balloon oesophagography and CT: case report [in Chinese]. Chin J Rehabil Med 32:1046-1047.
Anismus
Zhang Y, Wang ZN, He L, Gao G, Zhai Q, Yin ZT, Zeng XD (2014) (OS) Botulinum toxin type-A injection to treat patients with intractable anismus unresponsive to simple biofeedback training. World J Gastroenterol 20:12602-12607.
Raynaud's Syndrome
Zhao HM, Lian YJ (2015) (CS) Clinical and image improvement of Raynaud's phenomenon after botulinum toxin type A treatment. Austral J Dermatol 56:202-205.
Tinnitus
Liu HB, Fan JP, Lin SZ, Zhao SW, Lin Z (2011) (CS) Transient botulinum toxin A for tinnitus due to stapedius myoclonus: Case report. Clin Neurol Neurosurg 113:57-58.

Glandular Indications

Hyperhidrosis

Cao F, Zhang JD, Bai LM (2010) (OS) Efficacy of botulinum toxin A on osmidrosis: report of 42 cases [in Chinese]. Chin J Aesth Plast Surg 21:740-741.
Gao Y, Song J, Li H, Lu YG (2011) (RCT) High-dose botulinum toxin A injections for tretament of axillary hyperhidrosis [in Chinese]. Chin J Aesth Plast Surg 22:740-742.
Gao Y, Yang YD, Wang YY, Yang Y, Lu YG (2013) (IS) Efficacy of different doses of intradermal botulinum toxin A injections on axillary hyperhidrosis [in Chinese]. Chin J Med Aesth Cosmetol 19:100-102.
He JG, Wang T, Dong JS (2017) (OS) Efficacy of botulinum toxin A for the treatment of secondary axillary bromhidrosis. J Plast Reconstr Aesthet Surg 70:1641-1645.
He JG, Wang T, Dong JS (2018) (OS) A low initial botulinum toxin A treatment response does not predict poor long-term outcomes in patients with axillary bromhidrosis. J Dermatolog Treat 29:102-104.
Lin ZH, Gao YH, Liu JD (2016) (IS) Efficacy of two doses of botulinum toxin type A in the treatment of palmar hyperhidrosis [in Chinese]. Chin J Aesth Plast Surg 27:639-640.
Qi HL, Gu Q, Pan Y, Xu YC (2013) (OS) Botulinum toxin A for palmar hyperhidrosis [in Chinese]. Chin J Med Aesth Cosmetol 19:224-225.
Wang L, Gao H, Wei LY, Kong Q (2009) (OS) Treatment of axillary hyperhidrosis and osmidrosis with botulinum toxin A [in Chinese]. Chin J Med Aesth Cosmetol 15:173-175.
Wang T, Dong JS, He JG (2018) (OS) Long-Term safety and efficacy of botulinum toxin A treatment in adolescent patients with axillary bromhidrosis. Aesthetic Plast Surg 42:560-564.
Xie AG, Nie LJ, Tan Q (2014) (RCT) Local injections of botulinum toxin A: an alternative therapy for axillary osmidrosis. J Dermatol 41:153-156.
Zhu X, Zou X, Li ZJ, Liu SY, Zhao P (2012) (OS) Clinical application of botulinum toxin A for 30 cases of osmidrosis [in Chinese]. Chin J Aesthetic Med 21:1288-1289.

Sialorrhea

Peng GL, cai SY, Hu SX, Huang ZX, Wang YD, Cai HQ (2015) (RCT) The effect of botulinum toxin A combined with electric stimulation in the treatment of sialorrhea attributable to cerebral palsy [in Chinese]. Chin J Phys Med Rehabil 37:387-389.
You GQ, Liang HY, Liao L, Zhu HH, Cai YZ (2014) (OS) Botulinum toxin in the treatment of sialorrhea after brain damage：a preliminary study [in Chinese]. Chin J Phys Med Rehabil 36:936-938.
Zheng LX, Luo WF, Zhang QL, Hu WD, Li X, Dai YP, Liu CF (2011) (OS) Botulinum toxin A injections guided by ultrasound and electromyogram to treat severe sialorrhea [in Chinese]. Chin J Clin Neurosci 19:493-495.

Prostate Hyperplasia

Ding XD, Chen HX, Xiao HQ, Wang W, Ding ZG, Zhang GB, Fu DG (2015) (OS) Treatment of benign prostatic hyperplasia by ultrasound-guided botulinum toxin type A injection. Cell Biochem Biophys 73:357-359.

Pain Indications

Headache

Cheng AX, Wu S (2010) (IS) Safety and efficacy of botulinum toxin A in the treatment of chronic daily headache [in Chinese]. Chin J Neurol 43:874-877.
Cheng XL, Cheng XM (2006) (OS) Botulinum toxin A in 50 cases of episodic migraine [in Chinese]. J Zhengzhou Univ (Med Sci) 41:1004-1005.
Cui CW, Tang XM, Liu Q, Liu Q, Wang RF, Ye WC (2013) (OS) Hemodynamic study of botulinum toxin A in the treatment of tension headache [in Chinese]. Chin J Pract Nerv Dis 16:73-75.
Fu YG (2001) (OS) Botulinum toxin A on tension-type headache [in Chinese]. Clin Focus 16:584-585.
Ge LT, Du M, Wu HJ, Lin L, Zhou Y (2008) (RCT) Efficacy and safety of botulinum toxin A in treatment of severe migraine [in Chinese]. Neur Injury Funct Reconstr 3:434-435.
Gu YY, Feng LH (2016) (RCT) Efficacy of botulinum toxin A combined with infrared polarised light on chronic migraine [in Chinese]. Chin J Pract Nerv Dis 19:58-59.
Hu XY, Hu YY (2005) (OS) Botulinum toxin A for tension headache [in Chinese]. Chin J Phys Med Rehabil 27:342-345.
Hu YY, Hu XY (2005) (OS) Botulinum toxin A in migraine [in Chinese]. Chin J New Drugs Clin Rem 24:165-168.
Li SH, Xu ZL, Li JR, Tian T (2012) (RCT) Efficacy of botulinum toxin A in the treatment of migraine [in Chinese]. J Chin Phys 14:1469-1471.
Li XQ, Chen HS (2016) (OS) Efficacy and safety of botulinum toxin A therapy of chronic migraine [in Chinese]. Med Pharm J Chin PLA 28:43-46.
Lu QH, Xu JZ (2009) (RCT) Efficacy of botulinum toxin A on migraine [in Chinese]. Chin J Rehabil Med 24:277.
Lu ZH, Xie CC, Gao CR (2006) (OS) Treatment of headache with botulinum toxin A [in Chinese]. J Clin Anesthesiol 22:664-665.
Nie M, Zha TW (2006) (OS) Efficacy of botulinum toxin A in migraine [in Chinese]. Chin J Rehabil Theory Pract 12:541.
Shao YF, Zhang Y, Zhao P, Yan WJ, Kong XP, Fan LL, Hou YP (2013) (RCT) Botulinum toxin type a therapy in migraine: preclinical and clinical trials. Iran Red Crescent Med J 15:e7704.
Song JH, Ding XD, Xiao HQ, Huang L, Zhang GB (2012) (OS) Botulinum toxin A injection under colour ultrasound with water capsule guidance for treatment of chronic migraine [in Chinese]. J HBUM 31:25-28.
Song JH, Ding XD, Huang L, Hong Y, Chen HX, Zhang GB (2012) (RCT) Efficacy of botulinum toxin A combined with infrared polarised light for the treatment of chronic migraine [in Chinese]. Chin J Phys Med Rehabil 34:216-219.
Song JH, Zhang GB, Ding XD, Huang L, Hong Y, Chen HX (2015) (RCT) Efficacy of type a botulinum toxin injections and infrared polarized light on treating chronic migraine. Eur Rev Med Pharmacol Sci 19:1976-1982.
Wang JR, Zhu HY, Xu Y (2013) (RCT) Pericranial intramuscular injections of botulinum toxin A for treatment of chronic migraine [in Chinese]. Zhejiang Med J 35:38-40.Yuan ZM,
Yang HJ, Peng KR, Liu Y (2008) (IS) Efficacy of botulinum toxin A on episodic migraine [in Chinese]. Guangdong Med J 29:1396-1397.
Yue YH, Bai XD (2007) (OS) Clinical observations on 80 cases of primary headache treated by local injections of botulinum toxin A [in Chinese]. J Chin Phys 9:976-977.
Zhai YF, Zhang R, Wu HQ, Bu N, Wang HQ, Zhang GL (2017) (RCT) Botulinum toxin A for treatment of chronic tension headache [in Chinese]. Anhui Med Pharm J 21:914-617.
Zhang HW, Zhang HF, Wei YJ, Lian YJ, Chen Y, Zheng YK (2017) (OS) Treatment of chronic daily headache with comorbid anxiety and depression using botulinum toxin A: a prospective pilot

study. Int J Neurosci 127:285-290.

Trigeminal Neuralgia

Li S, Lian YJ, Chen Y, Zhang HF, Ma YQ, He CH, Wu CJ, Xie NC, Zheng YK, Zhang Y (2014) (OS) Therapeutic effect of botulinum toxin-A in 88 patients with trigeminal neuralgia with 14-month follow-up. J Headache Pain 15:43.
Liu J, Xu YY, Zhang QL, Luo WF (2018) (IS) Efficacy and safety of botulinum toxin type A in treating patients of advanced age with idiopathic trigeminal neuralgia. Pain Res Manag 2018:7365148.
Wang SY, Yue J, Xu YX, Xue LF, Xiao WL, Zhang CL (2014) (OS) Preliminary report of botulinum toxin A trigger point injections for treatment of trigeminal neuralgia： experiences in 16 cases [in Chinese]. Shanghai J Stomatol 23:117-119.
Wu CJ, Lian YJ, Zheng YK, Zhang HF, Chen Y, Xie NC, Wang LJ (2012) (RCT) Botulinum toxin type A for the treatment of trigeminal neuralgia: results from a randomized, double-blind, placebo-controlled trial. Cephalalgia 32:443-450.
Wu CJ, Xie NC, Liu HB, Zhang HF, Zhang L (2018) (CS) A new target for the treatment of trigeminal neuralgia with botulinum toxin type A. Neurol Sci 39:599-602.
Xia JH, He CH, Zhang HF, Lian YJ, Chen Y, Wu CJ, Ma YQ (2016) (OS) Botulinum toxin A in the treatment of trigeminal neuralgia. Int J Neurosci 126:348-353.
Xu YY, Zhang QL, Mao CJ, Hu WD, Zhou XP, Luo WF (2015) (IS) Efficacy and safety of botulinum toxin A in the treatment of idiopathic trigeminal neuralgia in patients older than 70 years [in Chinese]. Natl Med J China 95:1994-1996.
Zhang HF, Lian YJ, Ma YQ, Chen Y, He CH, Xie NC, Wu CJ (2014) (RCT) Two doses of botulinum toxin type A for the treatment of trigeminal neuralgia: observation of therapeutic effect from a randomized, double-blind, placebo-controlled trial. J Headache Pain. 15:65.
Zhang HF, Lian YJ, Xie NC, Chen C, Zheng YK (2017) (RCT) Single-dose botulinum toxin type A compared with repeated-dose for treatment of trigeminal neuralgia: a pilot study. J Headache Pain 18:81.

Postherpetic Neuralgia

Ding XD, Zhong J, Liu YP, Chen HX (2017) (OS) Botulinum as a toxin for treating post-herpetic neuralgia. Iran J Public Health 46:608-611.
Wang HM, Wang H, Liu FR, Chen ZJ (2013) (CS) Three cases of post-herpetic neuralgia treated with botulinum toxin A [in Chinese]. Clin Focus 28:927
Xiao LZ, Mackey S, Hui H, Xong DL, Zhang Q, Zhang DR (2010) (RCT) Subcutaneous injection of botulinum toxin a is beneficial in post-herpetic neuralgia. Pain Med 11:1827-1833.

Aesthetic Indications

Wrinkles

Primary Topic Publications

Chen JN, Li ZH, Lv XJ, Hu XC, Wang Y, Tian XC (2014) (OS) Short-term efficacy of botulinum toxin A for hyperkinetic facial lines [in Chinese]. J Tissue Eng Reconstr Surg 10:274-276.
Cheng XP (2003) (OS) Efficacy of botulinum toxin A for wrinkles in the elderly [in Chinese]. J Clin Dermatol 32:290-291.
Feng Z, Sun QN, He L, Wu Y, Xie HF, Zhao G, Xu JH, Yao C, Li HJ (2015) (RCT) Optimal dosage of botulinum toxin A for treatment of glabellar frown lines: efficacy and safety in a clinical trial. Dermatol Surg 41:S56-S63.
Guo XB, Zhang YX, Zhang Z, Jin R (2010) (OS) Botulinum toxin A in treating facial wrinkles [in Chinese]. J Tissue Eng Reconstr Surg 6:225-226.
Guo Y, Zhu SP, Shen JC, Wang AL, Deng L (2015) (OS) Quantitative evaluation of VISIA on Intense pulsed light combined with antiwrinkle injection treatment for facial skin aging [in Chinese]. Chin J Aesth Med 24:60-62.
He YH, Feng XH, Wei JH, Song XY, Liu GC (2008) (OS) Botulinum toxin A for treatment of dynamic wrinkles of the upper face [in Chinese]. Chin J Aesthetic Med 17:799-800.
Ke YH, Chen HB (2005) (OS) Botulinum toxin A for treatment of wrinkles in the upper third of the face [in Chinese]. Chin J Derm Venereol 19:698-699.
Kong YN (2001) (OS) Application of botulinum toxin A for facial wrinkles [in Chinese]. Chin J Aesthetic Med 10:204.
Li L, Li WX, Zhao J (2003) (OS) Botulinum toxin A for 58 cases of facial wrinkles [in Chinese]. China J Lepr Skin Dis 19:568-569.
Li M, Xu YZ (2009) (OS) Botulinum toxin A in the treatment of dynamic wrinkles of the upper face [in Chinese]. Chin J Aesth Plast Surg 20:170-172.
Li Q (2016) (RCT) Clinical study on the treatment of dynamic wrinkles of the upper face with different concentrations of botulinum toxin A [in Chinese]. Hebei Med J 22:549-552.
Li YM, Dong WW, Wang M, Xu N (2017) (IS) Investigation of the efficacy and safety of topical vibration anesthesia to reduce pain from cosmetic botulinum toxin A Injections in Chinese Patients: A multicenter, randomized, self-controlled Study. Dermatol Surg 43:S329-S335.
Li YP, Li QC, Liu YC (2005) (OS) Botulinum toxin A for facial wrinkles [in Chinese]. Chin J Pract Aesthet Plast Surg 16:105.
Liu Y, Li BB, Fang L, Zhang H, Duan HJ, Wang CJ, Yang MY (2013) (OS) Botulinum toxin A for treatment of facial wrinkles of the aging face [in Chinese]. Chin J Aesthetic Med 22:1026-1029.
Luo DH, Li BY, Zhou YH, Xu ZL, Li J (2009) (OS) Clinical observations on high-concentration botulinum toxin A therapy of facial wrinkles [in Chinese]. Chin J Aesth Plast Surg 20:465.
Ma LM, Liu Q (2009) (OS) Botulinum toxin A for treatment of wrinkles in the upper third of the face [in Chinese]. Chin J Aesthetic Med 18:1087-1088.
Ma Y, Li QY, Yan PX (2009) (OS) Treatment of dynamic facial wrinkles with botulinum toxin A [in Chinese]. Chin J Aesth Plast Surg 20:616-618.
Oliveira de Morais O, Matos Reis-Filho E, Vilela Pereira L, Martins Gomes C, Alves G (2012) (IS) Comparison of four botulinum neurotoxin type A preparations in the treatment of hyperdynamic forehead lines in men: a pilot study. J Drugs Dermatol 11:216-219.
Pan WL, Lu C, Tao XH (2010) (OS) Efficacy and safety of long-term treatment of facial wrinkles with botulinum toxin A [in Chinese]. Chin J Med Aesth Cosmetol 16:307-309.
Shang XX, Wu JD (2011) (OS) Injections of small amounts of botulinum toxin A in patients with wrinkles of the upper face [in Chinese]. Chin J Aesthetic Med 20:1705-1706.
Tao J, Fu ZL (2007) (OS) Botulinum toxin A for the treatment of 88 cases of facial wrinkles [in

Chinese]. Chin J Aesthetic Med 16:193-194.
Tian X, Li Y, Zhong WH, Gou QF, Chen F, Yao CY, Yang Z, Shao F (2009) (OS) Botulinum toxin A to treat 300 cases of facial wrinkles [in Chinese]. Chin J Aesthetic Med 18:161-162.
Wang Q, Liu DD, Hu Q (2007) (OS) Application of botulinum toxin A in 170 cases of facial wrinkles [in Chinese]. Chin J Misdiagn 7:5845-5846.
Wang XY, Zhu YF (2003) (OS) Botulinum toxin A against facial wrinkles [in Chinese]. Zhejiang Med J 25:509-510.
Wang Y, An HZ, Zhou XQ, Xiao HA (2018) (RCT) Efficacy and safety of low-dose botulinum toxin A on dynamic cross striation [in Chinese]. Chin J Aesth Plast Surg 29:165-168.
Wang Y, Liu XY, Tao K, Huang W, Zhang LP (2005) (OS) Botulinum toxin A for the treatment of wrinkles in the upper third of the face: report of 82 cases [in Chinese]. Chin J Pract Aesthet Plast Surg 16:9-10.
Xi GM, Huang CF, He GH, Li H (2004) (OS) Botulinum toxin A in 100 cases of facial wrinkles [in Chinese]. Chin J Med Aesth Cosmetol 10:50.
Xu Q (2005) (OS) Application of botulinum toxin A for wrinkles of frontal and temporal regions of the head [in Chinese]. Hebei Med J 11:1129.
Xi QC, Ning JL (2007) (OS) Repeated treatment of upper facial wrinkles with botulinum toxin A [in Chinese]. Anat Clin 12:58-59.
Yang WG, Yin Q (2002) (OS) Treatment of wrinkles of the upper half of the face with botulinum toxin A [in Chinese]. J Gansu Sci 14:74-77.
Yang WG, Yin Q (2003) (OS) Treatment of facial wrinkles with V-shaped symmetrical injections of botulinum toxin A [in Chinese]. J Pract Aesth Plasti Surg 14:86-88.
Zhang JD, Yang HD, Chen M, Zhang H, Xu PP (2012) (OS) Efficacy of low-volume botulinum toxin A in dynamic facial wrinkles [in Chinese]. Chin J Aesthetic Med 21:4-5.
Zhang KQ (2012) (OS) Efficacy and safety of repeated injections of botulinum toxin A in facial wrinkles [in Chinese]. Chin J Aesthetic Med 21:2-3.
Zhang YJ, Lin XL (2013) (OS) The effect of botulinum toxin A on dynamic and static wrinkles of the face [in Chinese]. JGCTCM 35:217-218.
Zhong YL, Xu J, Cheng BZ, Yu B, Deng YF (2004) (OS) Botulinum toxin A in reducing facial wrinkles: clinical observations in 100 cases [in Chinese]. China J Lepr Skin Dis 20:12-14.
Zhu J, Ji X, Xu Y, Liu J, Miao YY, Zhang JA, Luo D, Zhou BR (2017) (RCT) Efficacy of intradermal injections of botulinum toxin A for facial rejuvenation. Dermatol Ther 30:e12433.
Zhu L, Wu YF, Tang YL (2014) (RCT) Comparison of two dilutions of botulinum toxin A in the treatment of dynamic facial wrinkles [in Chinese]. Chin J Aesth Plast Surg 25:517-560.
Secondary Topic Publications
Deng YF, Wang CL (2002) (OS) Botulinum toxin A in Meige syndrome and facial wrinkles [in Chinese]. Chin J Dermatol 35:393-395.

Calf Reduction

Chen B, Gao ZW, Ma L, Ren C, Qi HJ, Qi YW, Lu H (2017) (OS) Application of botulinum toxin A in cosmetic calf contouring [in Chinese]. Chin J Aesthetic Med 26:51-52.
Li B, Tan J, li GF, Zhu Y (2010) (OS) Application of botulinum toxin A in calf reduction [in Chinese]. Chin J Aesthetic Med 19:976-977.
Xia W, Liu ZF, Han DM, Zhao Q, Zhao L, Ma Y (2014) (OS) Treatment of gastrocnemius muscle hypertrophy with botulinum toxin A [in Chinese]. Chin J Aesth Plast Surg 25:4-6.
Yao H, Li SR, Wang XM, Wu JL, Wang L, Liu JY (2013) (IS) Comparison of two different methods for calf reduction [in Chinese]. J Third Mil Med Univ 35:2231-2233.
Zhang K, Lv QF (2014) (OS) Reshape the calf by small doses of botulinum toxin A [in Chinese]. Chin J Aesthetic Med 23:1148-1150.

Zhang RH, Luo LJ, Xia XL, Shao H, Lv QF (2006) (OS) Reconstructing the leg by liposuction and injection of botulinum toxin A [in Chinese]. Chin J Aesth Plast Surg 17:353-354.

Masseter Reduction

Xie Y, Zhou J, Li HZ, Cheng C, Herrler T, Li QF (2014) (OS) Classification of masseter hypertrophy for tailored botulinum toxin A treatment. Plast Reconstr Surg 134:209e-218e.

Zhao JZ, Yang PY, Liu WG, Dai Q (2000) (OS) Treatment of masseteric hypertrophy with botulinum toxin A [in Chinese]. Chin J Med Aesth Cosmetol 6:63-64.

Scars

Hu L, Zou Y, Chang SJ, Qiu YJ, Chen H, Gang M, Jin YB, Lin XX (2018) (IS) Effects of botulinum toxin A on improving facial surgical scars: a prospective, split-scar, double-blind, randomized controlled trial. Plast Reconstr Surg 141:646-650.

Xiao ZB, Zhang FM, Cui ZW (2009) (OS) Treatment of hypertrophic scars with intralesional botulinum toxin type A injections: a preliminary report. Aesthetic Plast Surg. 33:409-412.

Acne

Zhou JF, Lu X (2009) (OS) Clinical observations of botulinum toxin A for 28 cases of acne [in Chinese].Med Inf 22:259.

Methods

Drug Comparison

Jiang HY, Chen S, Zhou J, Leung KK, Yu P (2014) (IS) Diffusion of two botulinum toxins type A on the forehead: double-blinded, randomized, controlled study. Dermatol Surg 40:184-192.

Jiang HY, Chen S, Zhou J (2016) (IS) Diffusion comparison of two botulinum toxin A preparations in the forehead [in Chinese]. Chin J Med Aesth Cosmetol 22:150-153.

Tang XF, Wan XH (2000) (OS) Comparison of Botox with Chinese type A botulinum. Chin Med J (Engl) 113:794-798.

Secondary Topic Publications

Oliveira de Morais O, Matos Reis-Filho E, Vilela Pereira L, Martins Gomes C, Alves G (2012) (IS) Comparison of four botulinum neurotoxin type A preparations in the treatment of hyperdynamic forehead lines in men: a pilot study. J Drugs Dermatol 11:216-219.

Quagliato EM, Carelli EF, Viana MA (2010) (RCT) Prospective, randomised, double-blind study comparing botulinum toxins type a Botox and Prosigne for blepharospasm and hemifacial spasm treatment. Clin Neuropharmacol 33:27-31.

Tang XF, Wan XH, Huang G, Zhang QB, Li T (1999) (OS) The treatment of focal dystonia and muscle spasms with Botox and CBTX-A [in Chinese]. Chin J Neurol 32:135-138.

Remote Effects

Wan XH, Tang XF, Cui LY (1999) (IS) Remote effects of local injection of botulinum toxin A [in Chinese]. Acta Acad Med Sin 21:362-367.

Allergic Reactions

Careta MF, Delgado L, Patriota R (2015) (CS) Report of allergic reaction after application of botulinum toxin. Aesthet Surg J 35:NP102-NP105.

Yao JM, Zhao FJ (2008) (CS) Allergic reaction after application of botulinum toxin for anti-wrinkles: a case report. Chin J Aesth Plast Surg 19:394.

Reviews and Guidelines

Reviews

Ao LJ, Li YM (2005) (R) Recent advances of botulinum toxin type A for treatment of neurogenic bladder secondary to spinal cord injury [in Chinese]. Chin J Rehabil Med 20:855-857.
Chinese Dermatologist Association (2017) (R) Consensus on applications of botulinum toxin in cosmetic dermatology in China [in Chinese]. Chin J Aesth Med 26:3-8.
Dong SZ, Chen YJ, Li YR (2017) (R) Recent advances of botulinum toxin type A for treatment of upper limbs of cerebral palsy children [in Chinese]. Chin J Rehabil Med 32:966-969.
Du T, Lu G, Li YJ (2016) (R) Recent advances of botulinum toxin type A in chronic pain [in Chinese]. Chin J Pain Med 22:936-939
FeiYang HH, Luo Y, Zhang XD (2018) (R) A review of current parameter retention rate in assessing therapeutic effect of botulinum toxin A for aesthetic procedures [in Chinese]. Chin J Aesth Med 27:141-144.
Fu WJ, Mo ZN (2006) (R) Recent advances of botulinum toxin type A for treatment of overactive bladder [in Chinese]. J Clin Urol 21:234-237.
Fu WJ, Mo ZN (2010) (R) Recent advances of botulinum toxin type A for benign prostate hypertrophy [in Chinese]. J Clin Urol 25:791-793
Han Y, Stevens AL, Dashtipour K, Hauser RA, Mari Z (2016) (R) A mixed treatment comparison to compare the efficacy and safety of botulinum toxin treatments for cervical dystonia. J Neurol 263:772-780.
Li Y, Xie WY, Liu T, Luo WF (2018) (R) Recent advances in botulinum toxin for treatment of depression [in Chinese]. Natl Med J China 98:1531-1533.
Li YH, Liu JQ, Xiao D, Zhang W, Hu DH (2017) (R) Advances in the research of mechanism in prevention and treatment of scar with botulinum toxin type A and its clinical application [in Chinese]. Chin J Burns 33:254-256.
Li ZJ, Guo JP (2012) (R) Recent advances of botulinum toxin type A in sialorrhea disorders [in Chinese]. J Oral Sci Res 28:93-94.
Liu YY, Zhang TJ, Lei QS, Song CL (2017) (R) Clinical application of botulinum toxin type A in autonomic disorders [in Chinese]. J Apoplexy Nerv Dis 34:1042-1044.
Minimally Invasive Cosmetic Expert Group of Plastic Surgery Branch of Chinese Medical Association & Micro-plastics expert group of the Chinese Association of Integrative Medicine Board of Cosmetic Surgery (2016) (R) Consensus on applications of botulinum toxin in calf hypertrophy in China [in Chinese]. Chin J Aesth Plast Surg 28:6-7.
Nan XY, Li XD (2013) (R) Current applications of botulinum toxin type A in clinical urology [in Chinese]. J Mod Urol 18:315-318.
Tang CH, Gao CY (2015) (R) Recent advances in application of botulinum toxin type A for Neurological diseases [in Chinese]. J Clin Neurol 28:236-238.
Wang YC (2008) (R) Application of botulinum toxin in China and other countries [in Chinese]. J Microbes Infect 3:100-102.
Yu RY (2004) (R) Botulinum toxin A intradermal injection therapy for hyperhidrosis [in Chinese]. Chin J Derm Venereol 18:179-181.
Zhang LG, Li F (2015) (R) Recent advances in botulinum toxin type A for treatment of spasticity [in Chinese]. Chin J Phys Med Rehabil 37:475-477.

Guidelines

Chinese Association of Rehabilitation Medicine (2010, 2015) (G) Chinese guideline on botulinum toxin A in the treatment of adult limb spasticity [in Chinese]. Chin J Rehab Med 25:595-620, 30:81-110.
Minimally Invasive Cosmetic Expert Group of Plastic Surgery Branch of Chinese Medical

Association & Micro-plastics expert group of the Chinese Association of Integrative Medicine Board of Cosmetic Surgery (2016) (G) Clinical guideline on application of botulinum toxin A in plastic surgery [in Chinese]. Chin J Aesth Plast Surg 27(7):385-387.
